# Supplementary material for: Content-rich biological network constructed by mining PubMed abstracts
Source: BMC Bioinformatics. 2004 Oct 8;5:147. doi: 10.1186/1471-2105-5-147 (PMC528731; doi:10.1186/1471-2105-5-147)
Supplement: Additional File 2 — The original results of the above study (non-essential files are deleted to keep the file size under the limit set by BMC bioinformatics). [file 1471-2105-5-147-S2.bz2 › chilibotAdditionalFile2/dip10/286ID9571155E1111/html/BMI1_CBX2.html]

 


 **BMI1** and **CBX2** 
  
Found 12 abstracts in PubMed, retrieved 10.  
 

 What does Google say? 
 PDF only 
| .edu only 

---

**Interactive relationship** (e.g. stimulation, inhibition, etc)

**Neutral relationship**- We show that mice deficient for both  **bmi1**  and M33  [ **CBX2** ]  present stronger homeotic transformations of the axial skeleton as compared to each single Polycomb group mutant, indicating strong dosage interactions between those two genes.  Ref: 9716520 Development, 1998
- RAE28,  **BMI1**  and M33  [ **CBX2** ]  interact homotypically, and both RAE28 and M33  [ **CBX2** ]  interact with  **BMI1** , but not with each other.  Ref: 9571155 Biochem Biophys Res Commun, 1998
- M33  [ **CBX2** ]  and RAE28 coimmunoprecipitate and copurify as members of large complexes from F9 cells, which express  **BMI1**  at very low levels, suggesting that different Polycomb group complexes can form in different cells.  Ref: 9571155 Biochem Biophys Res Commun, 1998

**Non-interactive relationship** (e.g. studied together, co-existance, homology, etc.)

- Four mammalian homologues, mel 18, bmi 1  [ **BMI1** ] , M33  [ **CBX2** ]  and rae 28, are analyzed in this study.  Ref: 9809583 Mol Immunol, 1998
- Here we show that this PEV is influenced by the heterochromatic protein SUV39H1 and by the Polycomb group proteins M33  [ **CBX2** ]  and BMI 1  [ **BMI1** ] .  Ref: 10990462 EMBO J, 2000
- We show that mouse Polycomb group homologues, RAE28,  **BMI1**  and M33  [ **CBX2** ] , have overlapping but not identical expression patterns during embryogenesis and in adult tissues.  Ref: 9571155 Biochem Biophys Res Commun, 1998
- RAE28,  **BMI1** , and M33  [ **CBX2** ]  are members of heterogeneous multimeric mammalian Polycomb group complexes.  Ref: 9571155 Biochem Biophys Res Commun, 1998
- Gel filtration analysis of embryonic extracts indicates that RAE28,  **BMI1**  and M33  [ **CBX2** ]  exist in large multimeric complexes.  Ref: 9571155 Biochem Biophys Res Commun, 1998
- Mice deficient for homologues of the Drosophila PcG genes, such as M33  [ **CBX2** ] ,  **bmi1** , mel18, rae28 and eed, show altered Hox expression patterns.  Ref: 11044623 Mech Dev, 2000
- Here we discuss current insights into the role of mouse Pc G genes, with a particular focus on the best studied  **Bmi1** , Mel18 and M33  [ **CBX2** ]  genes, as representative examples.  Ref: 9487388 Cell Mol Life Sci, 1998
- Here we report the skeletal phenotypes of compound mutants for two Polycomb group genes  **bmi1**  and M33  [ **CBX2** ] .  Ref: 9716520 Development, 1998
